# Hu similarity coefficient: a clinically oriented metric to evaluate contour accuracy in radiation therapy

**DOI:** 10.1038/s41598-024-81167-7

**Published:** 2024-12-04

**Authors:** Harold Yang Hu, Shaw Yang Hu, Min Yang, Yanle Hu

**Affiliations:** 1 Basis Scottsdale, Scottsdale, AZ USA; 2 The George Washington University, Washington, DC USA; 3 Department of Radiation Oncology, Dignity Health, Phoenix, AZ USA; 4 Department of Radiation Oncology, Mayo Clinic in Arizona, 5881 East Mayo Boulevard, Phoenix, AZ 85054 USA

**Keywords:** Organ contouring, Quantitative Metric, Contour Accuracy, Radiation Therapy, Radiotherapy, Cancer imaging

## Abstract

To propose a clinically oriented quantitative metric, Hu similarity coefficient (HSC), to evaluate contour quality, gauge the performance of auto contouring methods, and aid effective allocation of clinical resources. The HSC is defined as the ratio of the number of boundary points of the initial contour that doesn’t require modifications over the number of boundary points of the final adjusted contour. To demonstrate the clinical utility of the HSC in contour evaluation, we used publicly available pelvic CT data from the Cancer Imaging Archive. The bladder was selected as the organ of interest. It was contoured by a certified medical dosimetrist and reviewed by a certified medical physicist. This contour served as the ground truth contour. From this contour, we simulated two contour sets. The first set had the same Dice similarity coefficient (DSC) but different HSCs, whereas the second set kept a constant HSC while exhibiting different DSCs. Four individuals were asked to adjust the simulated contours until they met clinical standards. The corresponding contour modification times were recorded and normalized by individual’s manual contouring times from scratch. The normalized contour modification time was correlated to the HSC and DSC to evaluate their suitability as quantitative metrics assessing contour quality. The HSC maintained a strong correlation with the normalized contour modification time when both sets of simulated contours were included in analysis. The correlation between the DSC and normalized contour modification time, however, was weak. Compared to the DSC, the HSC is more suitable for evaluating contour quality. We demonstrated that the HSC correlated well with the average normalized contour modification time. Clinically, contour modification time is the most relevant factor in allocating clinical resources. Therefore, the HSC is better suited than the DSC to assess contour quality from a clinical perspective.

**Supplementary Information:**

The online version contains supplementary material available at 10.1038/s41598-024-81167-7.

## Introduction

In radiation therapy, contouring the treatment target and surrounding organs-at-risk (OARs) is a crucial step in treatment planning. However, this process can be quite time-consuming^1–3^. To expedite contouring, various automatic methods based on atlas^1,4–8^ and deep learning^3,9–14^ have been developed and implemented in clinical practice. Despite these advancements, evaluating the performance of these automatic contouring method remains challenging^15^.

**Fig. 1:**
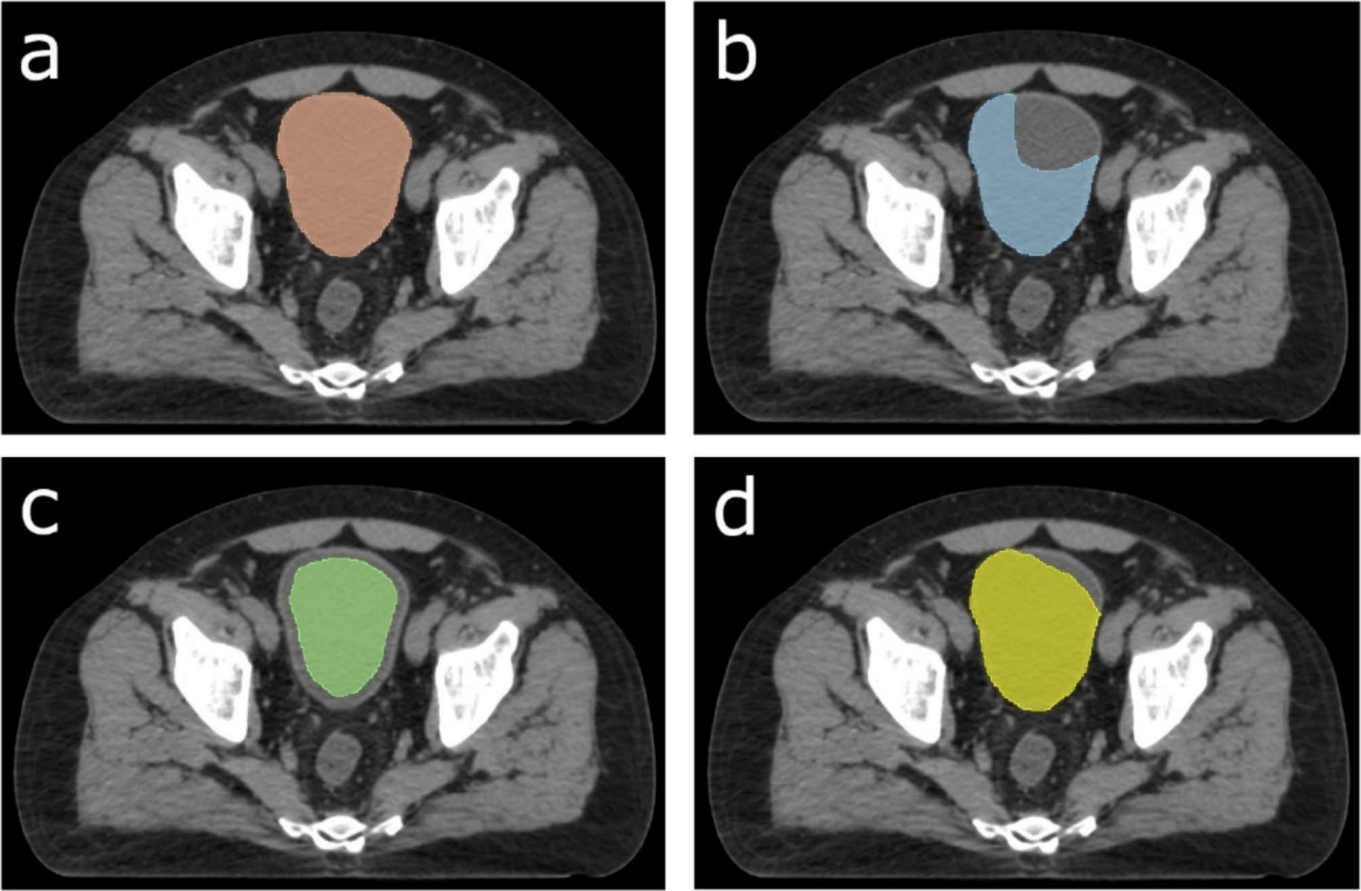
Illustration of the key component affecting the contour adjustment time. The key factor influencing the contour adjustment time is the amount of modification needed, rather than the overlap of the two contours. (a) represents the reference bladder contour. (**b**–**d**) are three simulated contours requiring adjustment. (**b**,**c**) share the same DSC, while (**b**,**d**) have an equal number of boundary points that don’t need to be modified. Since contouring is primarily focused on the boundary of the organ being contoured, (**c**) is essentially equivalent to contouring the entire bladder and thus requires more time than (**b**), despite the same DSC. (**b**) and (**d**) have different DSCs, but the amount of change (or number of boundary points requiring modification) is the same. Thus, the required times for contour adjustment remain similar.

Currently, commonly used quantitative metrics to evaluate contour quality are based on geometric similarity between the contour being evaluated and the ground truth contour, e.g., the Dice similarity coefficient (DSC)^15,16^. These metrics provide a quantitative assessment of geometric similarity between two contours. However, they fall short as reliable indicators of clinical efforts required to modify auto-generated contours into clinically acceptable ones^10^. For instance, two auto-generated contours may have the same DSC compared to the ground truth contour, yet the amount of time needed to refine them for clinical use can vary significantly. In other cases, two contours may exhibit significantly different DSCs, while the necessary contour modification times remain comparable (as shown in Fig. 1). In clinical practice, the required staff time is what is clinically meaningful and determines how much resource needs to be allocated to complete the contouring task.

The surface DSC^15,17,18^ and Added Path Length (APL)^18^ were initially proposed as clinically relevant metrics to quantify potential time savings in contour modification. While these metrics showed promise in initial explorations, they also had limitations that could hinder their adoption in clinical practice. Specifically, the surface DSC is defined as the overlap of two contours, normalized by the mean of the two contour surfaces. Since the unmodified (or raw) contour generated by auto-contouring methods is also used in the surface DSC calculation, it could disproportionately influence the surface DSC’s capability in predicting the time for contour adjustments. Vaassen et al.^18^ found that the correlation between the surface DSC and both the absolute contour modification time and relative time savings was not strong. The APL, defined as the path length needed to be added to comply with institutional contouring guidelines, measures the amount of manual modification required. Ideally, it should correlate well with the absolute contour modification time. Vaassen et al.^18^ did find an improved correlation between the APL and absolute contour modification time. However, using the APL as a metric to evaluate contour quality has its limitations. Firstly, it struggles to adequately handle inter-organ variability. Contour quality depends on both the size of the organ and the extent of modification needed for clinical acceptability. For the same APL, contour quality could be excellent for a large organ but poor for a small one. Secondly, the correlation with the absolute contour modification time could be subject to inter-observer variability, as it heavily depends on individual’s contouring habits and familiarity with specific contouring software. These factors ultimately dampen enthusiasm for adopting the APL in clinical practice.

To overcome these limitations, we propose a novel clinically oriented metric, termed the Hu similarity coefficient (HSC), to evaluate contour accuracy for radiation therapy. Instead of quantifying geometric similarity of two contours, the HSC focuses on quantifying the effort required for contour adjustments. It tracks the ratio of the boundary points that remain unchanged in the process of modifying an auto-generated contour to a clinically acceptable contour. In a DICOM RT Structure Set, contours are defined slice by slice using sequence of (x, y, z) triplets (or points) defining the boundary of a contour in the patient-based coordinate system^19^. During the contour adjustment process, if a boundary point is modified, its coordinates change accordingly. Thus, when comparing the initial and adjusted contours, the more boundary points remaining unchanged based on their coordinates, the less manual modification it requires to achieve the final contour. Ideally, a HSC of 1 signifies that no points are altered, indicating zero contour adjustment time. By normalizing the unchanged boundary point to the total number of boundary points in the final contour, it reduces the impact of inter-organ variability.

The clinical significance of the HSC is based on the hypothesis that contour adjustment time depends primarily on the number of boundary points that need to be modified. When adjusting the contour, the distance between a boundary point’s locations between the initial contour and clinically acceptable contour has minimal impact on the contour adjustment time. Instead, most contouring efforts focus on the edge of the organ being contoured. Notably, contouring software often includes a feature that automatically fills the space enclosed by the edge-defining contour once the organ’s edge has been delineated. As a result, the HSC has a great potential in offering a truly quantitative metric to gauge contour quality from a clinical perspective (i.e., required staff efforts in contour adjustments). It can provide valuable help aiding allocation of clinical resources.

To validate our hypothesis and demonstrate the potential of the HSC, we conducted a study examining the correlation between the HSC and normalized contour modification time. As a comparison, we also assessed the correlation between the DSC and normalized contour modification time. By normalizing the contour modification time to individual’s manual contouring time, it is possible to enable aggregation across multiple observers for comprehensive analysis.

## Results

Table 1 lists the calculated HSC and DSC for simulated contours, benchmarked against each individual’s final bladder contour. These values closely align with those benchmarked against the ground truth contour from which simulated contours were created. The minor differences observed are attributed to inter- and intra-observer variabilities. Notably, all these final bladder contours are clinically acceptable. They are indistinguishable from a clinical standpoint and therefore can be used in the calculation of the HSC and DSC.

**Table 1:** The calculated HSC and DSC based on individual observer’s final bladder contour.

Simulated contours	HSC (based on the final contour)					DSC (based on the final contour)				
	Obs. 1	Obs. 2	Obs. 3	Obs. 4	AVE	Obs. 1	Obs. 2	Obs. 3	Obs. 4	AVE
Set 1, Case 1	0.000	0.000	0.000	0.000	0.000	0.839	0.839	0.834	0.847	0.840
Set 1, Case 2	0.110	0.110	0.109	0.111	0.110	0.843	0.844	0.834	0.847	0.842
Set 1, Case 3	0.293	0.294	0.292	0.297	0.294	0.844	0.844	0.838	0.849	0.844
Set 1, Case 4	0.503	0.507	0.506	0.510	0.506	0.847	0.848	0.845	0.851	0.848
Set 1, Case 5	0.700	0.702	0.701	0.705	0.702	0.848	0.848	0.845	0.849	0.848
Set 2, Case 1	0.460	0.462	0.461	0.466	0.462	0.638	0.635	0.634	0.640	0.637
Set 2, Case 2	0.461	0.461	0.459	0.465	0.461	0.723	0.726	0.720	0.730	0.725
Set 2, Case 3	0.462	0.464	0.459	0.467	0.463	0.759	0.765	0.755	0.766	0.761
Set 2, Case 4	0.460	0.463	0.461	0.464	0.462	0.797	0.802	0.797	0.804	0.800
Set 2, Case 5	0.461	0.464	0.459	0.465	0.462	0.841	0.847	0.838	0.848	0.844
Set 2, Case 6	0.458	0.464	0.460	0.464	0.461	0.888	0.897	0.887	0.896	0.892
Set 2, Case 7	0.458	0.460	0.461	0.465	0.461	0.945	0.947	0.944	0.952	0.947

These values are essentially the same as those calculated based on the pre-determined pseudo ground truth contour (contour set #1: HSC = 0.000, 0.111, 0.295, 0.507 and 0.704, respectively, DSC = 0.850; contour set #2: HSC = 0.464, DSC = 0.640, 0.730, 0.765, 0.805, 0.849, 0.899, and 0.953, respectively). The minor differences are due to inter- and intra-observer variabilities.

Table 2 provides the times that were used to contour the bladder from scratch, and to adjust the simulated contours into clinically acceptable ones. There are large variations in absolute contour modification times among the four participants. For example, in Set 1, Case 1, the contouring time ranges from 385 s to 703 s (with a Max-to-Min ratio of 1.83). The significant variations pose challenges when aggregating data across multiple observers. The estimated contouring time based on the group data may deviate significantly from a given individual’s actual contouring time, making it clinically less meaningful. The inter-observer variations reduce dramatically when contour modification times were normalized by individual’s contouring time from scratch. For example, in Set 1, Case 1, the normalized contour modification time ranges from 0.917 to 1.017 (with a Max-to-Min ratio of 1.11). If there is a relationship between the normalized contour modification time and a specific quantitative metric, this relationship could be more accurately applied to individual cases.

**Table 2:** The absolute and normalized contour modification times for all observers.

Simulated contours	Contour modification time								
	Absolute (seconds)				Normalized				
	Obs. 1	Obs. 2	Obs. 3	Obs. 4	Obs. 1	Obs. 2	Obs. 3	Obs. 4	AVE
From scratch	730	420	631	584	-	-	-	-	-
Set 1, Case 1	703	385	642	575	0.963	0.917	1.017	0.985	0.970
Set 1, Case 2	601	293	591	551	0.823	0.698	0.937	0.943	0.850
Set 1, Case 3	517	227	465	366	0.708	0.540	0.737	0.627	0.653
Set 1, Case 4	350	180	365	283	0.479	0.429	0.578	0.485	0.493
Set 1, Case 5	256	119	233	200	0.351	0.283	0.369	0.342	0.336
Set 2, Case 1	369	156	407	300	0.505	0.371	0.645	0.514	0.509
Set 2, Case 2	356	181	405	296	0.488	0.431	0.642	0.507	0.517
Set 2, Case 3	375	198	406	287	0.514	0.471	0.643	0.491	0.530
Set 2, Case 4	366	180	399	304	0.501	0.429	0.632	0.521	0.521
Set 2, Case 5	378	197	399	291	0.518	0.469	0.632	0.498	0.529
Set 2, Case 6	371	200	403	297	0.508	0.476	0.639	0.509	0.533
Set 2, Case 7	367	220	405	303	0.503	0.524	0.642	0.519	0.547

Normalization is based on individual observer’s time to contour the bladder from scratch.

**Fig. 2:**
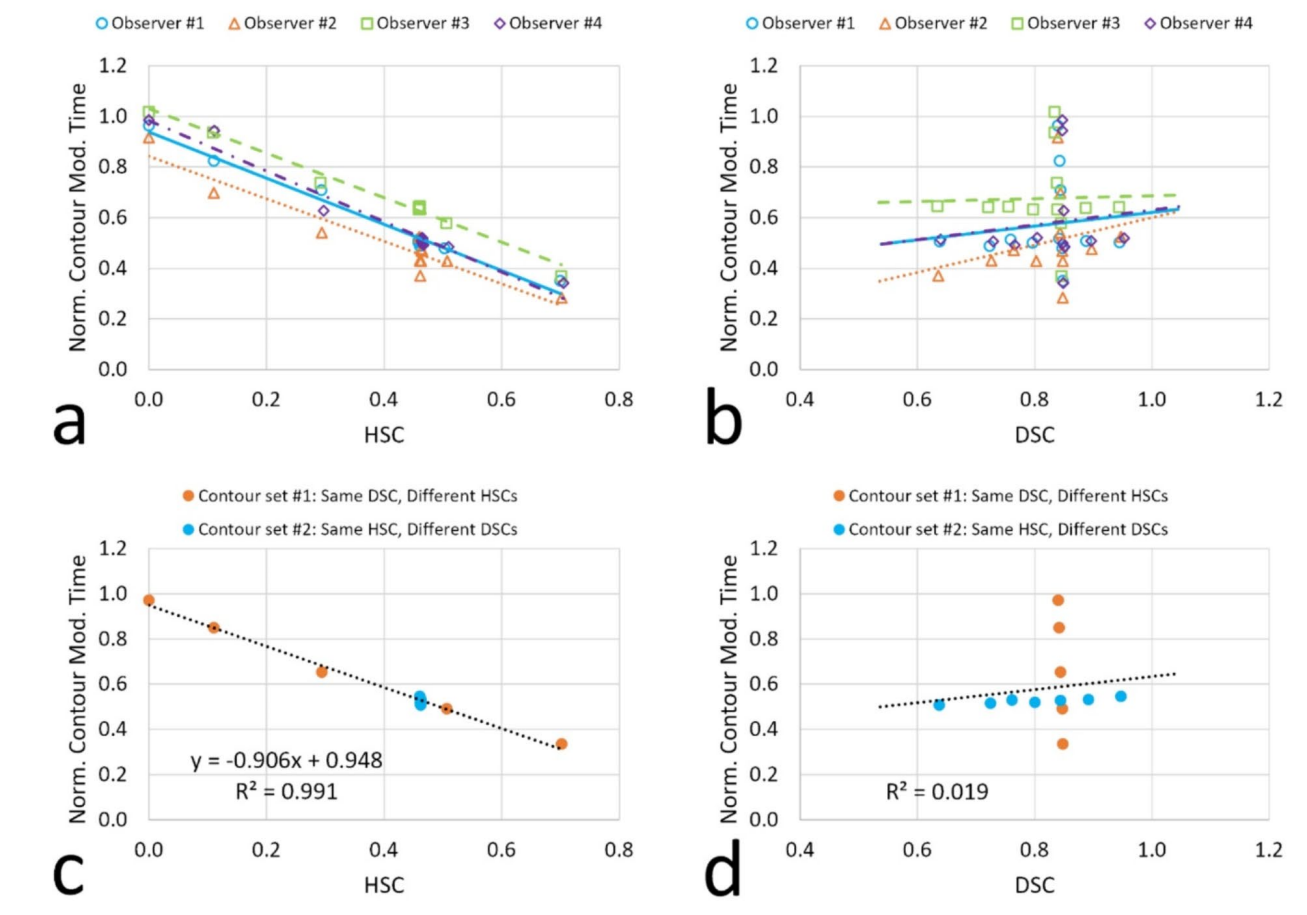
Correlations between the normalized contour modification time and two quantitative metrics (i.e., HSC and DSC). All simulated contours are included in analysis. (**a**,**b**) Plot the normalized contour modification time against the HSC and DSC, respectively for all four observers. (**c**,**d**) Plot the average normalized contour modification time against the average HSC and DSC, respectively. Normalization reduces inter-observer variability and makes aggregation of data feasible. A strong linear correlation is observed between the normalized contour modification time and the HSC for all observers (**a**). The linear correlation improves further when the average values are used, with an R^2^ value of 0.991 (**c**). Results from both simulated contour sets align well along the same fitting line. As a comparison, no obvious correlation is identified between the normalized contour modification time and the DSC (**b**,**d**), indicating inadequacy of using the DSC as an effective metric for assessing the clinical effort required during contour adjustment.

We plotted the normalized contour modification time against the HSC and DSC for all simulated contours combined from both contour sets. In Fig. 2a, it demonstrates a strong linear correlation between the normalized contour modification time and the HSC across all four observers (with R^2^ values of 0.979, 0.912, 0.983 and 0.965, respectively). The fitting lines exhibit a general parallelism to each other (slope = -0.913, -0.837, -0.879 and − 0.997, respectively), and noticeable differences in intercepts (0.939, 0.841, 1.030 and 0.984, respectively). The differences in intercepts likely stem from intra-observer variability, especially in the case of contouring the bladder from scratch which was used for normalization. For example, if an observer was slower in contouring the bladder from scratch but efficient in contouring for other cases, it could influence the fitting curve and result in an intercept less than 1. In Fig. 2b, no obvious correlation can be identified between the normalized contour modification time and DSC. Each observer’s data reveal two distinct groups: one aligned vertically (corresponding to the 1st contour set with a consistent DSC but varying HSCs) and another aligned horizontally (corresponding to the 2nd contour set with a consistent HSC but different DSCs). Figure 2b shows that it could require dramatically different times to adjust contours to meet clinical standards even though they might have very similar DSC (1st contour set), whereas the normalized contour modification time could remain similar for contours with significantly different DSCs (2nd contour set). In Fig. 2c, we plotted the average normalized contour modification time against the average HSC across all observers. The linear correlation between the two parameters improves further, with an R^2^ value of 0.991. Results from both simulated contour sets align well along the same fitting line. However, Fig. 2d reveal no obvious correlation between the average normalized contour modification time and the average DSC. The two simulated contour sets exhibit distinct patterns as those shown in Fig. 2b, indicating inadequacy of using the DSC as an effective quantitative metric for assessing the clinical effort required during contour adjustment.

## Discussion

In this work, we demonstrated that the HSC correlated strongly with the normalized contour modification time and therefore could be used as an effective quantitative metric to estimate contour quality from a clinically relevant perspective, i.e. required time for contour adjustment. When calculating the HSC, individual’s final contour, instead of the ground truth contour, is utilized to minimize the impact of intra- and inter-observer variabilities on assessing contour quality. In clinical practice, intra- and inter-observer variabilities always exist. As long as the final contours meet clinical standards, they are all acceptable and thus indistinguishable from a clinical perspective. The “ground truth” contour used in previous investigations is either supplied by one expert or agreed upon by multiple experts through a panel review. The traditional “ground truth” contour is not the true ground truth contour. It is best described as one of the clinically acceptable contours, or pseudo ground truth contours. For quantitative metrics based on geometric similarity, using a predetermined pseudo ground truth contour is less of an issue because these metrics are not sensitive to which pseudo ground truth contour is selected. But for the HSC, it does depend on the selection of the pseudo ground truth contour. In an extreme case in which all boundary points of the two contours for the same organ are offset by a small amount (e.g., 0.2 mm), the calculated HSC is essentially 0, even though both contours are clinically acceptable and require no modification at all. This is the reason we use individual’s final contour in the definition of the HSC. This proposed change enables us to focus more on the clinically relevant parameter, i.e., contour modification time. In this particular study, due to the great contrast of the bladder, all final contours remained clinically indistinguishable from the ground truth contour. The differences in the total number of boundary points between the final and ground truth contours were minor, as shown in Supplementary Table S1.

Calculation of the HSC is based on the clinically acceptable contour. How to determine if a contour is truly clinically acceptable, however, may be subject to debate. Different persons may have different standards based on their experience and their contouring habits. It is normal that one person deems a contour satisfactory whereas another person finds it less ideal and requires further adjustment. This is especially true when there is a lack of image contrast. Establishing an objective criterion for clinically acceptable contours may not be as straightforward as we think. In clinical practice, a relatively objective criterion is that if a contour is accepted in the subsequent physics and physician review, it can be considered as clinically acceptable. In our study, we selected bladder as the organ of interest to minimize contouring uncertainty since it has a great contrast compared to surrounding tissues on CT images. In addition, we presented the ground truth contour to all participants and asked them to practice contouring in 3D Slicer to gain familiarity with the software before performing contour adjustment tasks. These also helped reduce the confounding factors that might affect individual participant’s performance.

The simulated contours used in the study were specifically designed to demonstrate the advantages of the HSC, as well as inadequacy of the DSC, as a quantitative metric to evaluate contour quality. These two contour sets represent two rather extreme scenarios: contours with a similar DSC that may require dramatically different adjustment times to meet clinical standards, and contours with dramatically different DSCs that may need very similar time for adjustment. In real-world clinical cases, we encounter contours with various types and qualities. It is likely that we may observe certain correlation between the normalized contour modification time and DSC. But the correlation wouldn’t be very strong due to the inadequacy of the DSC definition. On the other hand, the HSC is defined based on the ratio of boundary points that do not need to be modified, which correlates well with the time required for contour adjustment. Therefore, it is expected that the HSC serves as a better quantitative metric for assessing the time required for contour adjustment. It holds a great potential in gauging contour quality and aiding effective allocation of clinical resources.

In the current study, the contouring software automatically fills the inner space once the edge is defined. This feature may not be offered in all contouring software. If it is not available, another viable solution is to use the post-processing function to fill all inner space (or all cavities). This one-step process applies to the entire contour on all slices and adds only a minor amount of time compared to contour adjustment. Therefore, it will not significantly impact the relationship between the HSC and normalized contour modification time. Additionally, all simulated contours used in this study were smaller than the final or ground truth contour. Theoretically, the initial contour could be either smaller or larger than the final contour. It seems to be two different scenarios. However, from the contouring perspective, they are quite similar. If the initial contour is smaller, we expend it to the final contour using the brush from the inside. Conversely, if the initial contour is larger, we push it to the final contour using the brush from the outside. The required effort remains similar when the amount of modification needed is comparable. Regardless of whether the initial contour is smaller or larger, the HSC is always less than or equal to 1, according to its definition. If the initial contour is larger, it may have more boundary points than the final contour, but only those that are also part of the final contour (untouched) are used in the HSC calculation, which is less than or equal to the total number of boundary points in the final contour.

Please note that the HSC is a more effective indicator of the required clinical effort when the initial contours are relatively smooth, regardless of whether modifications are needed on one side or in multiple separate locations. However, it has limitations and may become less effective in extreme scenarios where the contour oscillates at a high frequency (e.g., all odd boundary points are on the final contour, but all even boundary points are not). In such cases, the HSC’s usefulness is greatly reduced, as it would be easier to draw or brush through the entire boundary rather than only modifying the necessary portions. Fortunately, the initial contours from auto-contouring software are often smooth, making the HSC a good indicator of the effort required for contour modification.

We demonstrate that the HSC is an effective metric for evaluating contour quality from a clinical perspective. When multiple contours are created for the same organ using different methods, a higher HSC indicates better contour quality and relatively less contour editing time compared to a lower HSC. The HSC, however, is not intended to estimate the absolute time required for a specific staff member to either adjust a given contour to meet clinical standards or to contour an organ entirely from scratch. In clinical settings, the exact contouring time of a given staff member depends on many factors such as the size of the organ, the image contrast of the organ, contouring habits of the person completing the task, familiarity of a given staff member with contouring software, etc. Therefore, the HSC shall be used with caution. When introducing it into clinics, we need to consider potential confounding factors and set up appropriate expectations.

Auto-contouring tools have shown great potential in reducing the time required for contouring organs-at-risk^20^. However, adopting these tools in radiotherapy clinical practice is not straightforward and requires careful evaluation. Heilemann et al. proposed a comprehensive method for evaluating and implementing commercially available auto-contouring tools, emphasizing the importance of clinical acceptance^21^. They noted that existing quantitative metrics, such as the DSC and Hausdorff distance (HD), may not always correlate well with clinical ratings based on the effort required for contour adjustment (i.e., no modification, minor modification, major modification, and rejection). Clinical ratings provide only a coarse qualitative assessment with four categories, which can result in multiple auto-contouring tools falling into the same category (e.g., minor modification), making it difficult to determine which tool performs better. Our proposed metric, the HSC, addresses the limitations of qualitative clinical ratings by providing a detailed quantitative assessment. This metric can help determine the performance of auto-contouring tools more accurately, making it a powerful tool for implementing these tools in radiotherapy clinical practice.

The primary focus of the current study is to propose an effective quantitative metric to assess contour quality from a clinical perspective and aid allocation of clinical resources for contouring. The potential of the HSC, however, goes beyond these intended uses. With appropriate adaption, it may be used as a loss function to facilitate Artificial Intelligence (AI) model training. Given the fact that the denominator of the HSC is not unique and can be any of the pseudo ground truth contours (or clinically acceptable contours), it is a convenient quantitative metric when used retrospectively. When the HSC is used prospectively such as being a loss function in model training, it becomes complicated due to the non-unique characteristic of the pseudo ground truths. It is possible that an AI model may generate a contour that is very close to the predetermined ground truth contour, but all boundary points are slightly different. Clinically, the auto-generated contour is satisfactory and requires no further optimization. But the HSC based on a predetermined pseudo ground truth contour is low and prompts continuing optimization. To overcome this challenge, we can add some constraints to mimic human decision making. A practical constraint is to setup a limitation on geometric deviation. If a boundary point on the contour to be modified deviates from its position in the predetermined pseudo ground truth contour by an amount that is clinically indistinguishable, e.g., less than 0.5 mm, this boundary point is considered as clinically acceptable and treated as a boundary point requiring no modification when calculating the HSC. This practical adaptation enables us to integrate the HSC in model training and improve the performance of models from a clinical perspective by minimizing utilization of clinical resources. Utilizing the adapted HSC to facilitate model training, even though interesting, is out of the scope of the current study and will be further explored in future.

## Methods

### Definition of the HSC

**Fig. 3:**
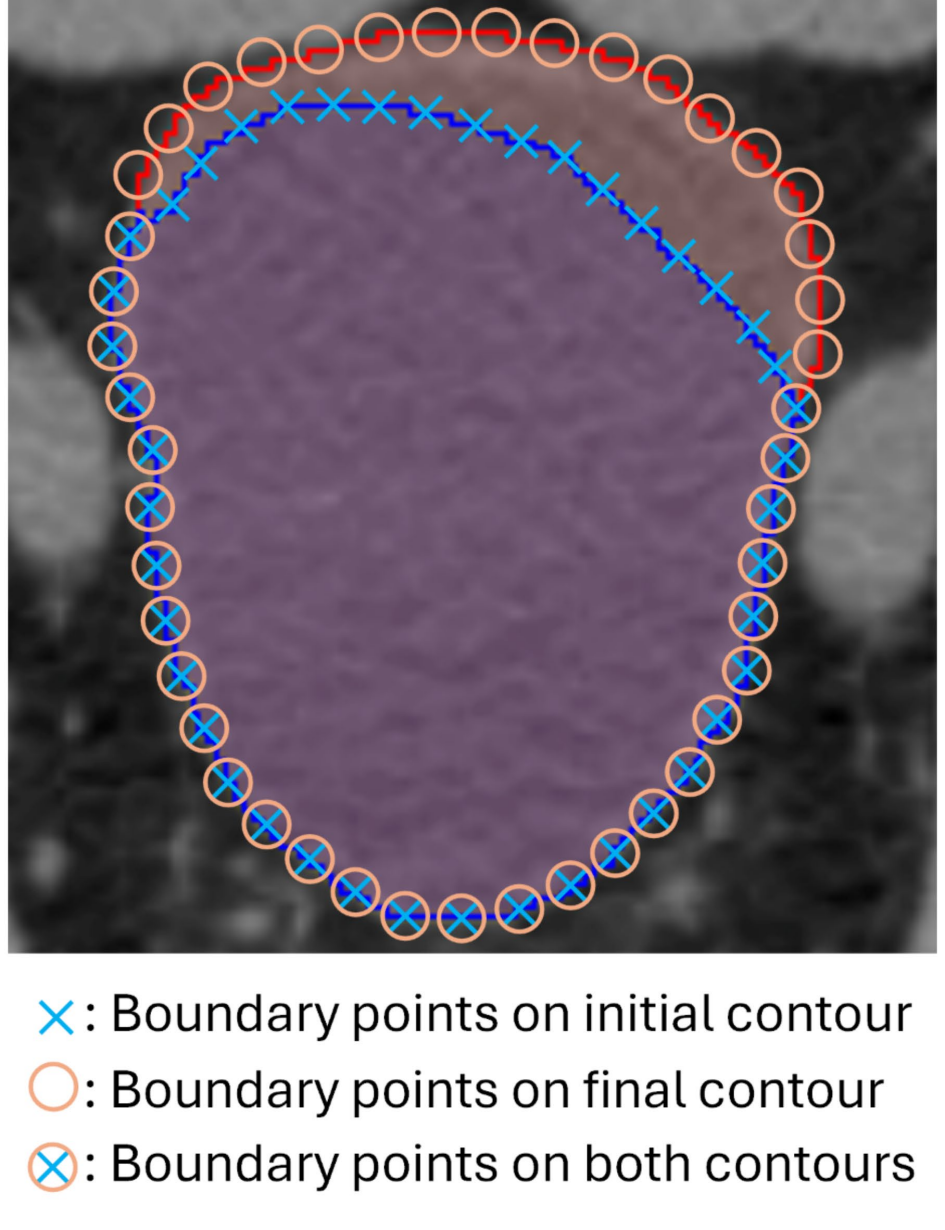
Illustration of the HSC definition, which is the ratio of boundary points shared by both the initial and final contours (marked by combined circle and cross symbols) to the total number of boundary points on the final contour (marked by circle symbols).

Figure 3 illustrates the definition of the HSC. The blue line represents the initial contour (boundary marked by cross symbols), whereas the red line represents the final contour after adjustment (boundary marked by circle symbols). Boundary shared by both the initial and final contours are marked by combined circle and cross symbols), indicating the portion that does not require modification. The HSC is defined as.

1 $$\:HSC={N}_{U}/{N}_{T}$$

where $\:{N}_{U}$ is the number of boundary points of the contour that doesn’t require modifications (combined circle and cross symbols) and $\:{N}_{T}$ is the total number of boundary points of the final contour after modification (circle symbols). Compared to other quantitative metrics, a notable distinction of the proposed HSC is that it is defined based on individual’s final contour, instead of the ground truth contour. In clinical practice, any final contours adhering to the institution’s contouring guidelines are considered clinically acceptable, with consensus contouring recommendations available (https://econtour.org/references). The term “ground truth contour” used in other metrics typically refers to one of these final contours, but it is not the only correct choice. Therefore, using individual’s final contour in the HSC makes it clinically more relevant.

Theoretically, the HSC ranges between 0 and 1. A HSC coefficient of 0 indicates that the entire contour needs to be modified to make it clinically acceptable. It is essentially equivalent to creating a contour from scratch. A HSC coefficient of 1 represents the ideal situation and means the entire contour can be used directly with no modifications.

### Image data and simulated contours

To demonstrate the clinical significance of the HSC in contour evaluation, we used publicly available pelvic CT data (Pelvic-Reference-Data, Pelvic-Ref-025) from the Cancer Imaging Archive^22^. The CT image set comprises 162 images with a slice thickness of 3 mm, an in-plane pixel size of 1 × 1 mm^2^, and an in-plane matrix size of 512 × 512. These images cover the entire pelvic region. Given images used in this study are anonymized and publically accessible, IRB review is not required. Among the various pelvic OARs, we specifically focused on the filled bladder due to its reasonable size, representative shape, and distinct contrast with surrounding tissues. This choice aimed to minimize inter- and intra-observer uncertainties associated with contouring.

**Fig. 4:**
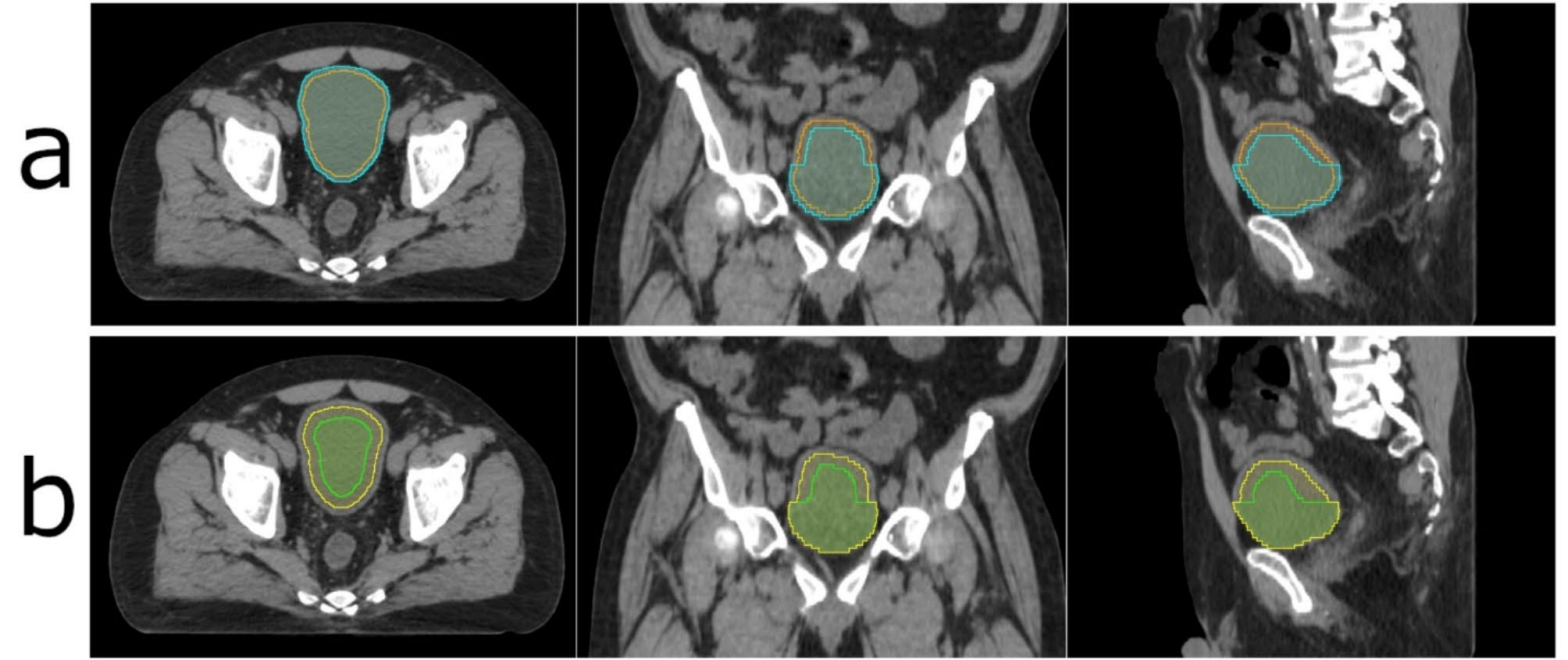
Demonstration of two simulated contour sets. (**a**) shows two simulated contours from the contour set #1 that have the same DSC (0.850) but different HSCs (0.000—Orange vs. 0.507—Cyan). (**b**) shows two simulated contours from the contour set #2 that have the same HSC (0.464) but different DSCs (0.765—green vs. 0.899—yellow).

Contouring of the bladder was performed in a free, open-source software called 3D Slicer (Ver 5.6.1, https://www.slicer.org/). The bladder was first contoured by a certified medical dosimetrist and then reviewed by a certified medical physicist. This bladder contour served as the ground truth contour. Based on the ground truth contour, two sets of contours were simulated. The first set included 5 contours (illustrated in Fig. 4a). It represented the scenario of the same DSC (0.850) but different HSCs (0, 0.111, 0.295, 0.507, and 0.704, respectively). This was achieved by increasing the number of slices in which the bladder contour remained untouched while progressively shrinking the bladder contour on the rest of slices to maintain the same DSC. The second set included 7 contours (illustrated in Fig. 4b). It simulated the scenario of the same HSC (0.464) but different DSCs (0.640, 0.730, 0.765, 0.805, 0.849, 0.899, and 0.953, respectively). This was achieved by keeping the bladder contour on a certain number of slices untouched while gradually shrinking the bladder contour on the rest of slices. When creating simulated contours, we calculated the DSC and HSC based on the ground truth contour of the bladder. The DSC was computed using a built-in function in 3D Slicer, while the HSC was determined with a custom Python script. Simulated contours were generated through a trial-and-error process. We adjusted the contours using 3D Slicer, calculated the DSC and HSC, and compared them to the target values. This adjustment process was repeated until the DSC and HSC matched the predetermined values. These simulated contours served as the starting point for our subsequent investigation into quantifying the time required for contour modifications.

### Contour modification time

Four independent observers, including a high school student, a college student, a board-certified medical physicist and a board-certified dosimetrist, participated in quantification of contour modification time. The high school student and college student were specifically trained for this project. They each have approximately six months of experience with 3D Slicer software and bladder contouring. The medical physicist has over 14 years of experience in clinical medical physics. The dosimetrist has more than 11 years of experience in clinical medical dosimetry. Prior to adjusting the simulated contours, all observers were presented with the ground truth bladder contour and asked to practice contouring the bladder from scratch till they were familiar with the software and their contours were deemed acceptable as-is by both certified dosimetrist and physicist. This practice aimed to minimize inter-observer variability arising from individual’s skills and familiarity with the contouring software.

After establishing familiarity with 3D Slicer (the contouring software), all observers were tasked with adjusting these simulated bladder contours into clinically acceptable bladder contours and recording the contour modification time for each case. Additionally, each observer was asked to contour the bladder from scratch and noted the time to finish the task. This time corresponded to the completely manual contouring time and was used to normalize that individual observer’s contour modification times. The normalized contour modification time facilitated the aggregation of data across multiple observers, allowing for a comprehensive analysis.

The normalized contour modification time was corelated with both the DSC and HSC. The suitability of using the DSC and HSC as indicators for the required clinical resource to complete the contouring task was evaluated. From a perspective of allocating clinical resources, a reliable indicator is expected to consistently correlate with the normalized contour modification time. Specifically, as the HSC or DSC increases, the normalized contour modification time should decrease consistently in all clinical scenarios, i.e., a combination of both sets of simulated contours. In this correlation analysis, the HSC and DSC were calculated based on individual’s final contours as those were exactly the final results, and all deemed clinically acceptable.

## Electronic supplementary material

Below is the link to the electronic supplementary material.

Supplementary Material 1

## Data Availability

The datasets and Python scripts produced in this study can be obtained from the corresponding author upon reasonable request.
