# Supplementary material for: Hu similarity coefficient: a clinically oriented metric to evaluate contour accuracy in radiation therapy
Source: Sci Rep. 2024 Dec 4;14:30215. doi: 10.1038/s41598-024-81167-7 (PMC11618765; doi:10.1038/s41598-024-81167-7)
Supplement: Supplementary file 1 — Supplementary Material 1 [file 41598_2024_81167_MOESM1_ESM.docx]

Supplementary Table S1: Differences in the total number of boundary points between the final modified contour and the ground truth contour.

| The total number of boundary points | | | | | | | | | |
| --- | --- | --- | --- | --- | --- | --- | --- | --- | --- |
| Simulated contour | Ground truth contour | Obs. 1 | | Obs. 2 | | Obs. 3 | | Obs. 4 | |
|  |  | Total # | Dev | Total # | Dev | Total # | Dev | Total # | Dev |
| Set 1, Case 1 | 8304 | 8434 | 1.6% | 8390 | 1.0% | 8444 | 1.7% | 8292 | -0.1% |
| Set 1, Case 2 | 8304 | 8346 | 0.5% | 8342 | 0.5% | 8458 | 1.9% | 8272 | -0.4% |
| Set 1, Case 3 | 8304 | 8356 | 0.6% | 8338 | 0.4% | 8396 | 1.1% | 8260 | -0.5% |
| Set 1, Case 4 | 8304 | 8378 | 0.9% | 8308 | 0.0% | 8336 | 0.4% | 8262 | -0.5% |
| Set 1, Case 5 | 8304 | 8350 | 0.6% | 8328 | 0.3% | 8334 | 0.4% | 8292 | -0.1% |
| Set 2, Case 1 | 8304 | 8374 | 0.8% | 8342 | 0.5% | 8360 | 0.7% | 8272 | -0.4% |
| Set 2, Case 2 | 8304 | 8358 | 0.7% | 8360 | 0.7% | 8394 | 1.1% | 8280 | -0.3% |
| Set 2, Case 3 | 8304 | 8342 | 0.5% | 8310 | 0.1% | 8388 | 1.0% | 8248 | -0.7% |
| Set 2, Case 4 | 8304 | 8382 | 0.9% | 8316 | 0.1% | 8364 | 0.7% | 8296 | -0.1% |
| Set 2, Case 5 | 8304 | 8364 | 0.7% | 8308 | 0.0% | 8394 | 1.1% | 8284 | -0.2% |
| Set 2, Case 6 | 8304 | 8410 | 1.3% | 8310 | 0.1% | 8378 | 0.9% | 8308 | 0.0% |
| Set 2, Case 7 | 8304 | 8410 | 1.3% | 8376 | 0.9% | 8362 | 0.7% | 8284 | -0.2% |
